# Current status and role of programmed ventricular stimulation in patients without sustained ventricular arrhythmias and reduced ejection fraction: Analysis of the Japan cardiac device treatment registry database

**DOI:** 10.1002/joa3.12468

**Published:** 2020-11-28

**Authors:** Hisashi Yokoshiki, Akihiko Shimizu, Takeshi Mitsuhashi, Kohei Ishibashi, Tomoyuki Kabutoya, Yasuhiro Yoshiga, Ritsuko Kohno, Haruhiko Abe, Akihiko Nogami, Members of the Implantable Cardioverter‐Defibrillator (ICD) Committee of the Japanese Heart Rhythm Society

**Affiliations:** ^1^ Department of Cardiovascular Medicine Sapporo City General Hospital Sapporo Japan; ^2^ UBE Kohsan Central Hospital Upe Japan; ^3^ Department of Cardiovascular Medicine Hoshi General Hospital Koriyama Japan; ^4^ Department of Cardiovascular Medicine National Cerebral and Cardiovascular Center Suita Japan; ^5^ Division of Cardiovascular Medicine Department of Medicine Jichi Medical University School of Medicine Shimotsuke Japan; ^6^ Division of Cardiology Department of Medicine and Clinical Science Yamaguchi University Graduate School of Medicine Ube Japan; ^7^ Department of Heart Rhythm Management University of Occupational & Environmental Health Kitakyushu Japan; ^8^ Cardiovascular Division Faculty of Medicine University of Tsukuba Tsukuba Japan

**Keywords:** cardiac resynchronization therapy with a defibrillator (CRT‐D), electrophysiological study (EPS), implantable cardioverter‐defibrillator (ICD), primary prevention, ventricular fibrillation (VF), ventricular tachycardia (VT)

## Abstract

**Background:**

The aim of this study was to clarify the current status and role of programmed ventricular stimulation in patients without sustained ventricular arrhythmias and reduced left ventricular ejection fraction (LVEF).

**Methods:**

The follow‐up data of the Japan cardiac device treatment registry (JCDTR) was analyzed in 746 patients with LVEF ≦35% and no prior history of sustained ventricular arrhythmias who underwent de novo implantable cardioverter‐defibrillator (ICD) or cardiac resynchronization therapy with a defibrillator (CRT‐D) implantation between January 2011 and August 2015.

**Results:**

Electrophysiological study (EPS) with programmed ventricular stimulation had been performed before the device implant in 118 patients (15.8%, EPS group). During the mean follow‐up of 21 ± 12 months, the rate of freedom from any death and appropriate defibrillator therapy was not significantly different between EPS group (n = 118) and No EPS group (n = 628). NYHA class II‐IV, and QRS duration were negatively associated with performing EPS. Among patients in the EPS group, the rate of ventricular tachycardia (VT)/ventricular fibrillation (VF) induction was 48%. The inducibility was not a predictor of appropriate defibrillator therapy, whereas BNP ≧535 pg/mL and no use of amiodarone were significantly associated with a risk of the appropriate therapy.

**Conclusion:**

EPS for induction of VT/VF had been performed in about 16% of patients with reduced LVEF before primary prevention ICD/CRT‐D implantation. Elevated BNP levels and no use of amiodarone, but not inducibility of VT/VF, appeared to be associated with appropriate defibrillator therapy in these populations.

## Graphical abstract

Electrophysiological study (EPS) had been performed with a rate of 15.8% in 746 patients with reduced ejection fraction (≦35%) who underwent primary prevention implantable cardioverter‐defibrillator (ICD) or cardiac resynchronization therapy with a defibrillator (CRT‐D) implant, based on the analysis of the Japan cardiac device treatment registry (JCDTR) database. Sustained ventricular arrhythmias were induced in about half of these patients, but the inducibility was not a predictor of appropriate defibrillator therapy. Elevated BNP levels and no use of amiodarone might be related to future appropriate defibrillator therapy in primary prevention ICD/CRT‐D patients with reduced ejection fraction (≦35%).

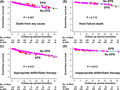

## 1 INTRODUCTION

Implantable cardioverter‐defibrillator (ICD) implantation, in combination with guideline‐directed medical therapy (GDMT), is an established therapy for primary prevention of sudden cardiac death in symptomatic heart failure with reduced ejection fraction. In the late 1990s and early 2000s, electrophysiological study (EPS) with programmed ventricular stimulation had been performed to identify patients at risk of ventricular arrhythmias,^1^, ^2^, ^3^ especially of an ischemic etiology,^1^, ^2^ whereas the results of randomized controlled trials underscored the use of ICDs for primary prevention of sudden cardiac death in patients with reduced left ventricular ejection fraction (LVEF) without performing the EPS.^4^, ^5^ Therefore, the significance of EPS in patients receiving the contemporary GDMT with reduced LVEF remains unknown.

The present study is aimed to evaluate the current status and significance of the EPS in patients with no prior history of sustained ventricular arrhythmias and LVEF ≦35% by analyzing the Japan cardiac device treatment registry (JCDTR) database.

## 2 METHODS

### 2.1 Study population

The JCDTR was established in 2006 by the Japanese Heart Rhythm Society (JHRS) for a survey of actual conditions in patients undergoing de novo implantation of Cardiac Implantable Electronic Devices (CIEDs) including implantable cardioverter‐defibrillator (ICD)/cardiac resynchronization therapy with a defibrillator (CRT‐D)/cardiac resynchronization therapy with a pacemaker (CRT‐P).^6^, ^7^, ^8^, ^9^ A new system, called New JCDTR, started on January 2019, in which data of patients at the implantation date after January 2018 are encouraged to register (https://membnew.jhrs.or.jp/newjcdtr/ accessed on March 1, 2020). The protocol for this research project has been approved by a suitably constituted Ethics Committee of each institution and it conforms to the provisions of the Declaration of Helsinki.

In general, the electrophysiologic testing protocol used 400‐ms and 600‐ms drive trains followed by one to three ventricular extrastimuli and rapid burst pacing from the right ventricular apex and then from the right ventricular outflow tract. Extrastimuli were decremented down to a coupling interval no shorter than 180 ms.^10^ In cases where no sustained ventricular tachyarrhythmia was induced, the protocol was repeated during isoproterenol challenge with the discretion of the attending physician. A sustained ventricular arrhythmia was defined as one lasting 30 seconds or requiring termination sooner because of hemodynamic compromise.

The present study analyzed the data of patients having a defibrillator (ICD or CRT‐D) with LVEF ≦35% and no prior history of sustained ventricular arrhythmias whose implantation date was from January 2011 to August 2015. Among them, the follow‐up data were available in 746 patients as of 16 September 2015. These 746 patients were analyzed in the present study.

### 2.2 Device programming

In general, device programming was as follows. VF zone detected ventricular events faster than 185‐200 beats/min with at least one train of anti‐tachycardia pacing (ATP) before shock, and the VT zone detected ventricular events faster than 150‐170 beats/min with at least three trains of ATP before shock. After the multicenter automatic defibrillator implantation trial—reduce inappropriate therapy (MADIT‐RIT) trial was published in 2012,^11^ the VF zone ≧200‐250 beats/min with ATP plus shock and VT zone ≧170 beats/min with delayed therapy (a 60‐second delay) or only monitoring were recommended. The discrimination algorithms were used at the physician's discretion.

### 2.3 Outcomes

The analyzed events were (a) death from any cause, (b) heart failure death, (c) appropriate and inappropriate defibrillator therapies. Appropriate defibrillator therapy was defined as an anti‐tachycardia pacing or shock for tachyarrhythmia determined to be either ventricular tachycardia (VT) or ventricular fibrillation (VF). The diagnosis of the cause of death was made by attending physicians.

### 2.4 Statistical analysis

All data are expressed as mean ± SD. Simple between‐group analysis was conducted using Student's *t*‐test. Categorical variables were compared using Fisher's exact test. Kaplan‐Meier curves were constructed to estimate event‐free outcomes in the two study groups with comparison using the log‐rank test. A logistic regression analysis was used to estimate the factors associated with performing EPS before CIEDs implant. Among the variables that reached a significance level of *P* < .1 in univariate models, multivariate analysis was performed. In patients who underwent EPS, a multivariate Cox proportional‐hazards regression model with a stepwise selection was used to estimate significant factors for appropriate defibrillator therapy. The sensitivity and specificity of BNP levels for the prediction of appropriate defibrillator therapy were evaluated using receiver operating characteristic (ROC) curve. Differences with *P* < .05 were considered significant. Statview version 5.0 for Windows (SAS Institute Inc) or R software ver.3.6.3 (https://www.r‐project.org/) was used for all statistical analyses.

## 3 RESULTS

### 3.1 Patient characteristics

Among 746 patients with LVEF ≦35% and no prior history of sustained ventricular arrhythmias, 118 patients (15.8%) underwent EPS with programmed ventricular stimulation before the ICD/CRT‐D implant, and 628 patients did not. The characteristics of patients with EPS (n = 118; EPS group) and those with no EPS (n = 628; No EPS group) are shown in Table 1. These data were derived from the status of each patient just before the device implantation. With regard to gender and the etiology of heart disease, there was no difference between the two groups. In No EPS group, age was higher, LVEF was lower, NYHA class was worse, and QRS duration was longer than those in EPS group. There was a significant increase in the percentage of CRT‐D implantation in No EPS group (79.3%) as compared to EPS group (46.6%). Patients in No EPS group had a lower history of non‐sustained ventricular tachycardia (NSVT) than those in EPS group. BNP and creatinine were higher and hemoglobin was lower in No EPS group than in EPS group.

**TABLE 1:** Characteristics of patients with and without EPS

	EPS (n = 118)	No EPS (n = 628)	*P* value
Age (y)	63.4 ± 12.0	66.7 ± 11.1	.0032
Male	93 (78.8)	488 (77.7)	.791
Underlying heart disease			.149
Ischemic	47 (39.8)	207 (33.0)	
Non‐ischemic	71 (60.2)	421 (67.0)	
LVEF (%)	26.1 ± 6.2	24.8 ± 6.5	.0498
NYHA class			<.0001
I	14 (11.9)	24 (3.8)	
II	50 (42.4)	184 (29.3)	
III	51 (43.2)	359 (57.2)	
IV	3 (2.5)	61 (9.7)	
Heart rate (/min)	70.6 ± 15.6	71.7 ± 16.3	.499
QRS duration (ms)	132.4 ± 31.7	146.9 ± 34.2	<.0001
QT interval (ms)	437.9 ± 55.1	448.5 ± 53.5	.0506
Device			<.0001
ICD	63 (53.4)	130 (20.7)	
CRT‐D	55 (46.6)	498 (79.3)	
Atrial lead			.314
Absent	15 (12.7)	103 (16.4)	
Present	103 (87.3)	525 (83.6)	
NSVT^a^	54 (85.7)	183 (68.3)	.0058
AF	14 (11.9)	82 (13.1)	.723
Diabetes mellitus	34 (28.8)	210 (33.4)	.326
Hypertension	53 (44.9)	253 (40.3)	.348
Dyslipidemia	41 (34.7)	198 (31.5)	.492
Hyperuricemia	22 (18.6)	136 (21.7)	.463
Cerebral infarction	12 (10.2)	49 (7.8)	.389
Peripheral artery disease	6 (5.1)	17 (2.7)	.170
BNP (pg/mL)^b^	525.6 ± 557.6	780.7 ± 1324.8	.0528
Log BNP^b^	5.74 ± 1.10	6.08 ± 1.09	.0033
Hemoglobin (g/dL)^c^	13.4 ± 2.1	12.7 ± 2.1	.0014
Creatinine (mg/dL)^d^	1.22 ± 1.10	1.51 ± 1.51	.0447

Note

Values are means ± SD, or number (%).

Abbreviations: AF, atrial fibrillation; LVEF, left ventricular ejection fraction; NSVT, non‐sustained ventricular tachycardia.

^a^ Information regarding the presence or absence of NSVT was available in 63 patients with EPS and 268 patients without EPS.

^b^ The value of BNP was not available in 13 patients with EPS and 73 patients without EPS.

^c^ The value of hemoglobin was not available in 8 patients without EPS.

^d^ The value of creatinine was not available in 1 patient with EPS and 14 patients without EPS.

Pharmacological therapy in EPS and No EPS groups is shown in Table 2. Use of beta blockers and angiotensin converting enzyme inhibitor (ACEI) or angiotensin II receptor blocker (ARB) was lower in No EPS group vs EPS group. The rate of having diuretics was higher in No EPS group than in EPS group.

**TABLE 2:** Pharmacological therapy in patients with and without EPS

	EPS (n = 118)	No EPS (n = 628)	*P* value
Ia	1 (0.8)	5 (0.8)	.954
Ib	5 (4.2)	19 (3.0)	.552
Ic	1 (0.8)	5 (0.8)	.954
β‐blockers	101 (85.6)	485 (77.2)	.042
III	38 (32.2)	207 (33.0)	.872
Ca^2+^ antagonists	14 (11.9)	59 (9.4)	.408
Digitalis	9 (7.6)	83 (13.2)	.090
Diuretics	78 (66.1)	508 (80.9)	.0003
ACEI/ARB	88 (74.6)	418 (66.6)	.087
Aldosterone antagonists	53 (44.9)	283 (45.1)	.976
Nitrates	10 (8.5)	70 (11.1)	.389
Statins	40 (33.9)	201 (32.0)	.687
Oral anticoagulant agents	54 (45.8)	317 (50.5)	.347
Antiplatelet agents	55 (46.6)	262 (41.7)	.324

Note

Data are given as number (%).

Abbreviations: ACEI, angiotensin converting enzyme inhibitor; ARB, angiotensin II receptor blocker.

### 3.2 Outcomes

During a mean follow‐up period of 21 ± 12 months, death from any cause occurred in 17 of 118 patients (14.4%) in EPS group and 111 of 628 patients (17.6%) in No EPS group. These events included 9 heart failure death (7.8%), 2 sudden cardiac death (1.7%) and 6 non‐cardiac death (5.1%) in EPS group and 51 heart failure death (8.1%), 12 sudden cardiac death (1.9%) and 48 non‐cardiac death (7.6%) in No EPS group.

**FIGURE 1:**
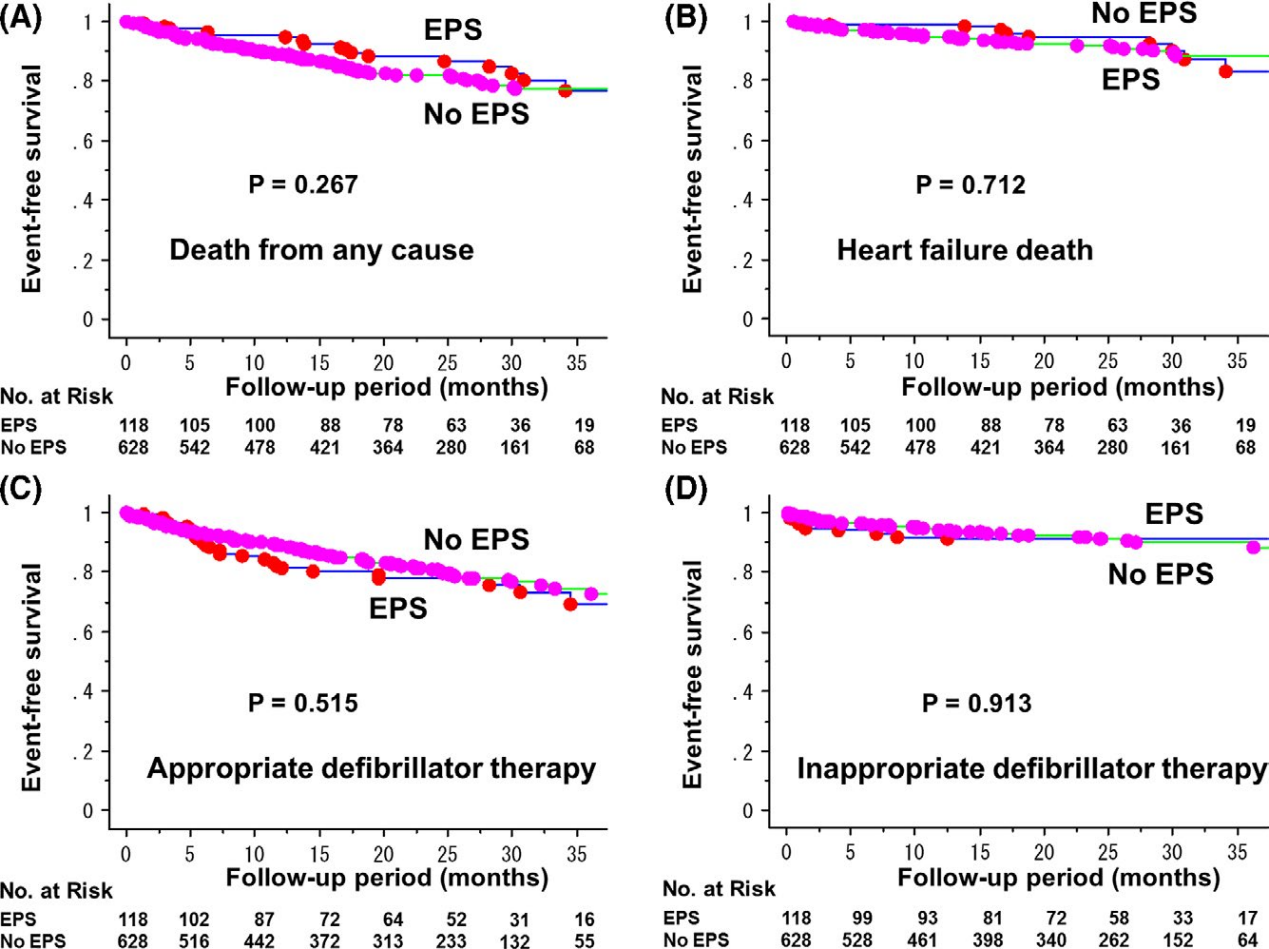
Kaplan‐Meier estimates of event‐free survival in ICD/CRT‐D recipients for primary prevention of sudden cardiac death with and without performing EPS before the implantation. Outcome events were death from any cause (A), heart failure death (B), appropriate defibrillator therapy (C) and inappropriate defibrillator therapy (D)

Kaplan‐Meier estimates of event‐free survival in the two groups are shown in Figure 1. The rate of event free survival for death from any cause was 95.5% at 1‐year and 88.2% at 2‐year in EPS group, and 89.2% at 1‐year and 82.0% at 2‐year in No EPS group (*P* = .267) (Figure 1A). Similarly, with regard to heart failure death, there was no significant difference between the two groups (Figure 1B). The rate of appropriate defibrillator therapy (shock and/or anti‐tachycardia pacing) was 18.8% at 1‐year and 22.3% at 2‐year in EPS group, and 11.2% at 1‐year and 19.1% at 2‐year in No EPS group (*P* = .515) (Figure 1C). There was no significant difference in the risk of inappropriate defibrillator therapy between the two groups (Figure 1D). Analyses separately performed between the patients with ischemic and non‐ischemic etiology revealed similar results in terms of death from any cause (Figure S1. non‐ischemic [A], ischemic [B]) and appropriate defibrillator therapy (Figure S1. non‐ischemic [C], ischemic [D]).

The variables associated with not performing EPS before the ICD/CRT‐D implantation obtained by multivariate models were NYHA class II‐IV (*P* = .030), and QRS duration (*P* = .002) (Table 3).

**TABLE 3:** Logistic regression analyses for factors associated with performance of EPS in patients with primary prevention and LVEF of ≦35%

	Univariate			Multivariate		
	OR	95% CI	*P* value	OR	95% CI	*P* value
Age (y)	0.975	0.959‐0.992	.0035			
LVEF	1.031	1.000‐1.063	.0504			
NYHA class II‐IV	0.295	0.148‐0.589	.0005	0.398	0.173‐0.917	.030
QRS duration (ms)	0.987	0.980‐0.993	<.0001	0.989	0.983‐0.996	.002
Cr (mg/dL)	0.794	0.628‐1.004	.0538			
Hemoglobin (g/dL)	1.169	1.061‐1.288	.0016			
BNP (pg/mL)	1.000	0.999‐1.000	.0226			

Abbreviations: 95% CI, 95% confidence interval; LVEF, left ventricular ejection fraction; OR, odds ratio.

### 3.3 Subgroup analysis of patients with EPS

Sustained VT or VF was induced by programmed ventricular stimulation in 57 patients (48%) of the EPS group (VT/VF induction) and was not induced in the remaining 61 patients (52%) (No VT/VF induction). In patients with VT/VF induction, the ratio of ischemic etiology was higher (49.1% vs 31.1%, *P* = .046) and the heart failure symptom assessed by NYHA class appeared to be less severe (*P* = .091), as compared to those with No VT/VF induction (Table 4). With regard to the pharmacological therapy, there was an increase in the use of statins (43.9% vs 24.6%, *P* = .027) and antiplatelet agents (61.4% vs 32.8%, *P* = .0018) in the VT/VF induction group vs the No VT/VF induction group (Table 5).

**TABLE 4:** Characteristics of patients with and without VT/VF induction by EPS

	VT/VF induction (n = 57)	No VT/VF induction (n = 61)	*P* value
Age (y)	63.9 ± 12.9	62.9 ± 11.1	.648
Male	41 (71.9)	52 (85.2)	.076
Underlying heart disease			.046
Ischemic	28 (49.1)	19 (31.1)	
Non‐ischemic	29 (50.9)	42 (68.9)	
LVEF (%)	26.8 ± 5.9	25.4 ± 6.3	.219
NYHA class			.091
I	11 (19.3)	3 (4.9)	
II	24 (42.1)	26 (42.6)	
III	21 (36.8)	30 (49.2)	
IV	1 (1.8)	2 (3.3)	
Heart rate (/min)	68.4 ± 13.5	72.7 ± 17.1	.140
QRS duration (ms)	128.1 ± 30.6	136.3 ± 32.4	.162
QT interval (ms)	438.6 ± 56.5	437.3 ± 54.2	.894
Device			.187
ICD	34 (59.6)	29 (47.5)	
CRT‐D	23 (40.4)	32 (52.5)	
Atrial lead			.214
Absent	5 (8.8)	10 (16.4)	
Present	52 (91.2)	51 (83.6)	
NSVT^a^	30 (88.2)	24 (82.8)	.535
AF	5 (8.8)	9 (14.8)	.315
Diabetes mellitus	19 (33.3)	15 (24.6)	.294
Hypertension	28 (49.1)	25 (41.0)	.374
Dyslipidemia	24 (42.1)	17 (27.9)	.104
Hyperuricemia	13 (22.8)	9 (14.8)	.261
Cerebral infarction	6 (10.5)	6 (9.8)	.901
Peripheral artery disease	4 (7.0)	2 (3.3)	.355
BNP (pg/mL)^b^	561.2 ± 601.6	494.5 ± 519.5	.543
Log BNP^b^	5.80 ± 1.11	5.68 ± 1.09	.597
Hemoglobin (g/dL)	13.2 ± 2.1	13.6 ± 2.1	.308
Creatinine (mg/dL)^c^	1.37 ± 1.44	1.08 ± 0.62	.148

Note

Values are means ± SD, or number (%).

Abbreviations: AF, atrial fibrillation; LVEF, left ventricular ejection fraction; NSVT, non‐sustained ventricular tachycardia.

^a^ Information regarding the presence or absence of NSVT was available in 34 patients with VT/VF induction and 29 patients without VT/VF induction.

^b^ The value of BNP was not available in 8 patients with VT/VF induction and 5 patients without VT/VF induction.

^c^ The value of creatinine was not available in 1 patient with VT/VF induction.

**TABLE 5:** Pharmacological therapy in patients with and without VT/VF induction by EPS

	VT/VF induction (n = 57)	No VT/VF induction (n = 61)	*P* value
Ia	1 (1.8)	0 (0.0)	.298
Ib	2 (3.5)	3 (4.9)	.704
Ic	1 (1.8)	0 (0.0)	.298
β‐blockers	50 (87.7)	51 (83.6)	.524
III	21 (36.8)	17 (27.9)	.297
Ca^2+^ antagonists	9 (15.8)	5 (8.2)	.202
Digitalis	2 (3.5)	7 (11.5)	.103
Diuretics	38 (66.7)	40 (65.6)	.900
ACEI/ARB	45 (78.9)	43 (70.5)	.291
Aldosterone antagonists	23 (40.3)	30 (49.2)	.335
Nitrates	7 (12.3)	3 (4.9)	.151
Statins	25 (43.9)	15 (24.6)	.027
Oral anticoagulant agents	25 (43.9)	29 (47.5)	.688
Antiplatelet agents	35 (61.4)	20 (32.8)	.0018

Note

Data are given as number (%).

Abbreviations: ACEI, angiotensin converting enzyme inhibitor; ARB, angiotensin II receptor blocker.

**FIGURE 2:**
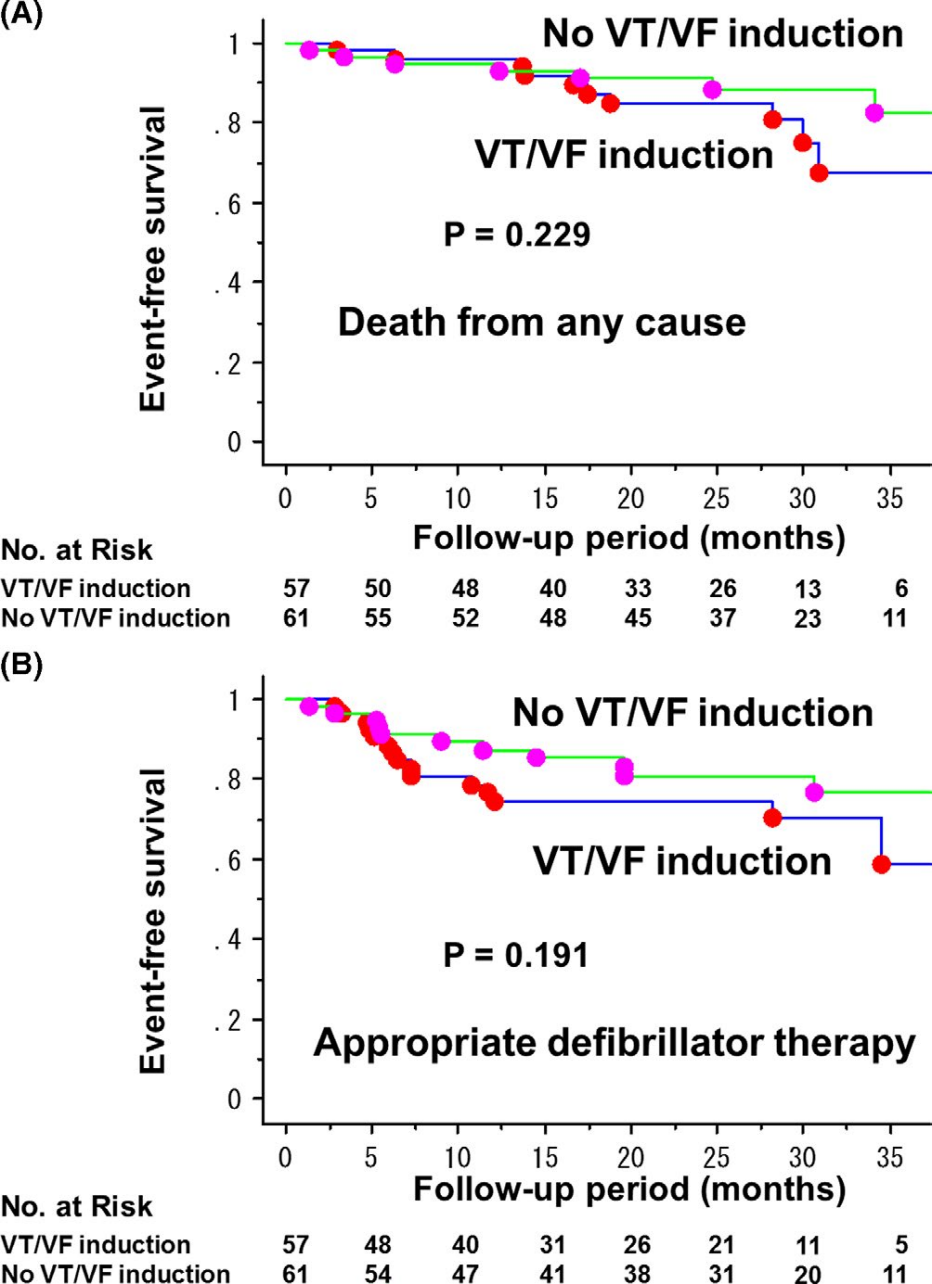
Kaplan‐Meier estimates of event‐free survival in ICD/CRT‐D recipients for primary prevention of sudden cardiac death with and without VT/VF induction. Outcome events were death from any cause (A), and appropriate defibrillator therapy (B)

The rate of event free survival for death from any cause was 96.2% at 1‐year and 84.9% at 2‐year in VT/VF induction group, and 94.9% at 1‐year and 91.1% at 2‐year in No VT/VF induction group (*P* = .230) (Figure 2A). Appropriate defibrillator therapy occurred in 26 patients (22.0%). The rate was 23.4% at 1‐year and 25.5% at 2‐year in VT/VF induction group, and 12.6% at 1‐year and 18.9% at 2‐year in No VT/VF induction group (*P* = .191) (Figure 2B). The event free survivals for death from any cause and appropriate defibrillator therapy were analyzed separately in the patients with non‐ischemic (Figure S2A,C) and ischemic (Figure S2B,D). However, there were no significant differences in the rate of these events between the two groups with or without VT/VF induction.

**FIGURE 3:**
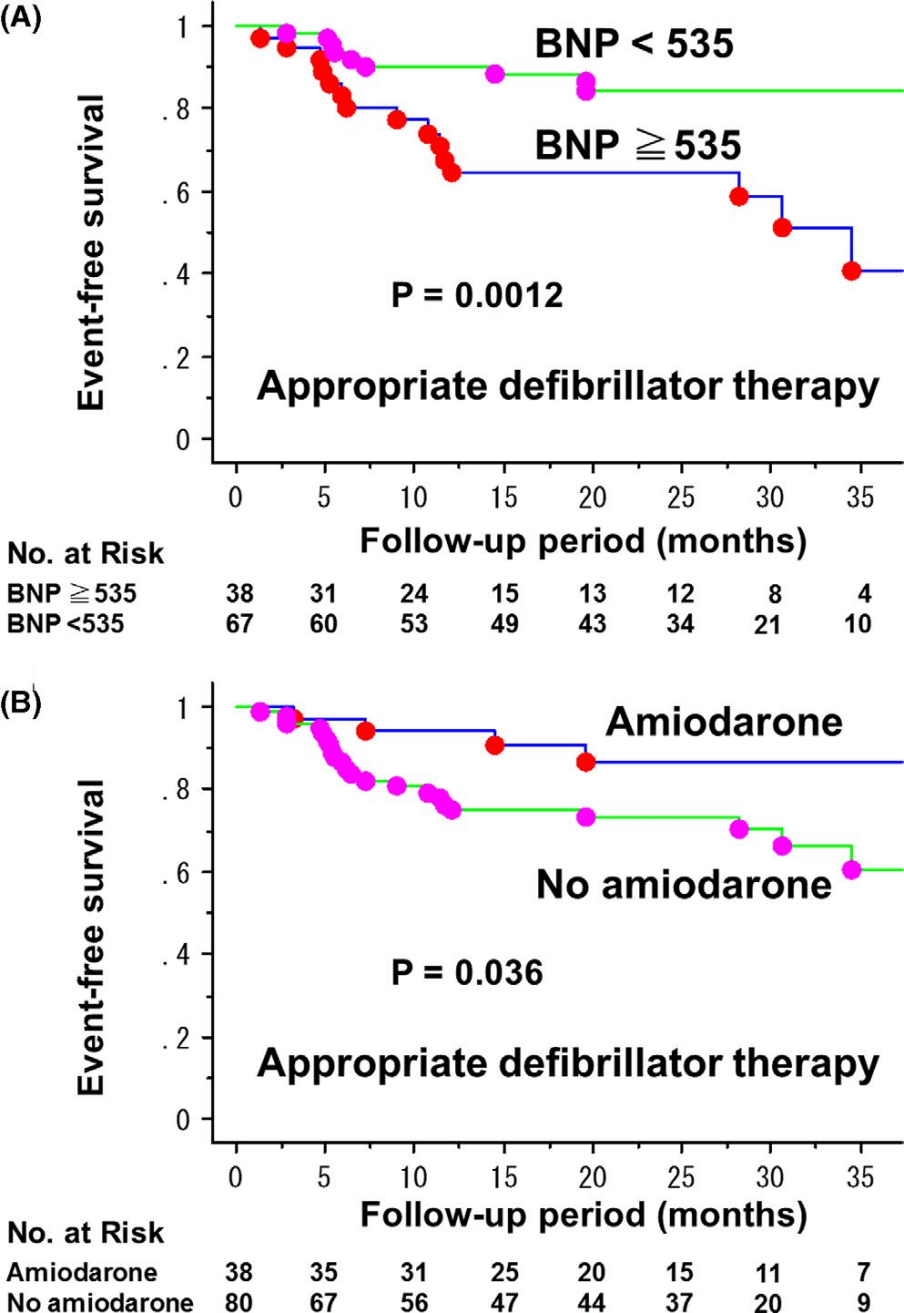
Kaplan‐Meier estimates of freedom from appropriate defibrillator therapy among ICD/CRT‐D recipients for primary prevention of sudden cardiac death who underwent EPS, stratified by BNP levels (A) and amiodarone use (B)

The variables associated with a risk of appropriate defibrillator therapy obtained by univariate models (*P* < .05) were QRS duration (*P* = .032), increase in baseline BNP levels (*P* = .0006), and no use of class III antiarrhythmic drugs (*P* = .046). Inducibility of VT/VF by programmed ventricular stimulation, NYHA class and LVEF were not significantly associated with the risk. A multivariate Cox proportional‐hazards regression model with stepwise selection method identified increase in BNP levels and no use of class III antiarrhythmic drugs as significant risk factors for appropriate defibrillator therapy. Thirty eight patients (32.2%) had class III antiarrhythmic drugs, all of which was amiodarone. The cut‐off level of BNP was 534.7 pg/mL which was determined ROC curve analysis with the area under curve of 0.63. The rate of appropriate defibrillator therapy was 32.4% at 1 year in patients with BNP ≧535 and 9.8% at 1 year in those with BNP <535 (*P* = .0012) (Figure 3A). It was 5.6% at 1 year in patients with amiodarone and 23.7% at 1 year in those without amiodarone (Figure 3B).

## 4 DISCUSSION

In patients with coronary artery disease, LVEF ≦40%, and asymptomatic NSVT, the inducibility of sustained ventricular tachyarrhythmias was associated with a significantly higher risk of sudden death or cardiac arrest.^1^ More recently, induction of VT/VF during programmed ventricular stimulation was an independent prognostic factor for future appropriate ICD therapy in patients with nonischemic cardiomyopathy and no history of spontaneous VT/VF.^12^ However, the predictive value of ventricular arrhythmia inducibility for subsequent VT/VF was not significant in patients with a LVEF ≦30% (or 35%).^2^, ^3^, ^10^ For example, among patients with coronary artery disease and LVEF <30%, the percentage of deaths that were arrhythmic was not significantly different in those with inducible tachyarrhythmia vs those without (55% vs 48%, *P* = .20).^2^ To the best of our knowledge, the present study has demonstrated for the first time in a contemporary cohort study, (a) the inducibility was not a predictor of appropriate defibrillator therapy in patients with reduced LVEF (≦35%) and (b) the rate of sustained VT/VF inducibility was 48% in those patients. The high inducibility (41%^10^ and 38%^3^) had been reported in patients whose LVEF was ≦30% or ≦35%.

According to the indication of health insurance in Japan, induction of VT/VF is generally required to approve ICD implantation for primary prevention of sudden cardiac death regardless the severity of left ventricular dysfunction and coexistence of symptomatic heart failure. This is because the indication was issued in 1996 based on the 1990 guideline of the Japanese Heart Rhythm Society and remains unchanged despite randomized control trials for primary prevention ICD implantation.^4^, ^5^ The 2018 guideline of the Japanese Circulation Society/Japanese Heart Rhythm Society on Non‐Pharmacotherapy of Cardiac Arrhythmias recommends primary prevention ICD implant as class IIa in ischemic or nonischemic patients with symptomatic heart failure, LVEF ≦35%, and a history of NSVT despite receiving contemporary care with GDMT. The present study demonstrated, with the analysis of the JCDTR database, induction of VT/VF with EPS had been performed with a rate of 15.8% before primary prevention ICD/CRT‐D implantation in patients with reduced ejection fraction (≦35%). Two factors, NYHA class II‐IV and QRS prolongation which indicate a candidate for CRT‐D implant, were associated with not performing the EPS. It may be time for the current indication of health insurance to be revised according to the latest guideline.

There were some differences of basic characteristics and pharmacological therapy between patients with and without performing EPS (Tables 1 and 2). For example, the use of β‐blockers was lower despite worse NYHA‐class and lower LVEF. Since the use of β‐blockers in patients receiving a CRT‐D was about 80% and it did not differ between non‐ university and university hospitals,^13^ the clinical practice appears to be homogeneous in facilities participating in the JCDTR. Significant risk factors for failing a trial of β‐blocker therapy in patients with chronic heart failure were worse NYHA status and worse left ventricular function.^14^ The former was a predictor of not performing EPS (Table 3), and can be a reason for paradoxical decrease in the rate of β‐blocker therapy in No EPS group.

In patients with prior myocardial infarction, sustained VT/VF and LVEF ≧35%, amiodarone was not inferior to ICD implantation with regard to the survival benefit.^15^ Mortality in patients with nonischemic cardiomyopathy, LVEF ≦35%, and asymptomatic NSVT who were treated with amiodarone or an ICD were not statistically significant in the Amiodarone vs Implantable Cardioverter‐Defibrillator Trial (AMIOVIRT).^16^ Amiodarone in combination with β‐blockers was effective for preventing ICD shocks compared with β‐blocker alone in patients with inducible or spontaneous occurring VT/VF.^17^ These results are in agreement with our observation that amiodarone significantly reduced the risk of appropriate defibrillator therapy in primary prevention ICD/CRT‐D patients. However, we should be prudent for the use of amiodarone, because it had no effect on all‐cause mortality in patients with symptomatic heart failure (NYHA class II or III) and LVEF ≦35%,^4^ except an increase in non‐cardiac mortality in NYHA class III.^18^

Elevated BNP levels were superior to EPS for predicting future appropriate defibrillator therapy (Figure 3A). Elevated baseline and follow‐up BNP levels were reported to be independent predictors of increased risk for subsequent VT/VF in symptomatic heart failure patients enrolled in MADIT‐CRT.^19^ The present study underscore this finding with an cutoff value of baseline BNP≧535 pg/mL for the prediction. Measurement of BNP is a simple, less invasive, and less expensive test compared with EPS, thus may be a useful marker of VT/VF in patients with reduced LVEF.

### 4.1 Study limitations

There are several limitations to be considered in this study. First, the protocol for programmed ventricular stimulations may not be uniform among facilities participating in the JCDTR. However, the physicians participating in the JCDTR must have a license approved by the JHRS which gives several lectures and an examination. Thus, the participants are qualified for performing EPS appropriately. Second, the use of isoproterenol during programmed ventricular stimulations depended on the discretion of the attending physicians, and the data regarding with and without isoproterenol provocation were unavailable. Third, there may be confounding factors with regard to the relationship between use of amiodarone and occurrence of VT/VF. Fourth, elevated BNP levels were identified as a predictor of appropriate defibrillator therapy in the subgroup of patients alone who underwent EPS (n = 118). We could not find any significant predictor in all the patients (n = 746) enrolled in the present study. As patients without EPS received a CRT‐D with a rate of 79.3% (Table 1), reverse remodeling with CRT may change the heart failure status and reduce the incidence of VT/VF.^20^, ^21^ Fifth, information regarding the presence or absence of NSVT, which is likely to be a surrogate marker of severe heart failure,^22^ is not mandatory for the registration of data in the JCDTR. The second version of JCDTR (New JCDTR) is now operative (https://membnew.jhrs.or.jp/newjcdtr/) and is prospectively collecting data including a history of NSVT from all the patients undergone ICD/CRT‐D/CRT‐P implantation after January 2018.

### 4.2 Conclusions

Inducibility of VT/VF by EPS, which had been performed in 15.8% of patients with LVEF ≦35% before primary prevention ICD/CRT‐D implantation in Japan, was not a significant predictor of subsequent appropriate defibrillator therapy. Patients with symptomatic heart failure (NYHA class II‐IV) and QRS prolongation, which indicate prerequisites for a CRT candidate, are unlikely to receive the EPS. Elevated BNP levels ≧535 pg/mL may be useful for predicting future VT/VF events in patients receiving primary prevention ICD/CRT‐D.

## CONFLICT OF INTEREST

All authors declare no conflict of interest related to this study.

## Supporting information

Fig S1

Fig S2
