# Supplementary figures and images for: Current status and role of programmed ventricular stimulation in patients without sustained ventricular arrhythmias and reduced ejection fraction: Analysis of the Japan cardiac device treatment registry database
Source: J Arrhythm. 2020 Nov 28;37(1):148–56. doi: 10.1002/joa3.12468 (PMC7896472; doi:10.1002/joa3.12468)

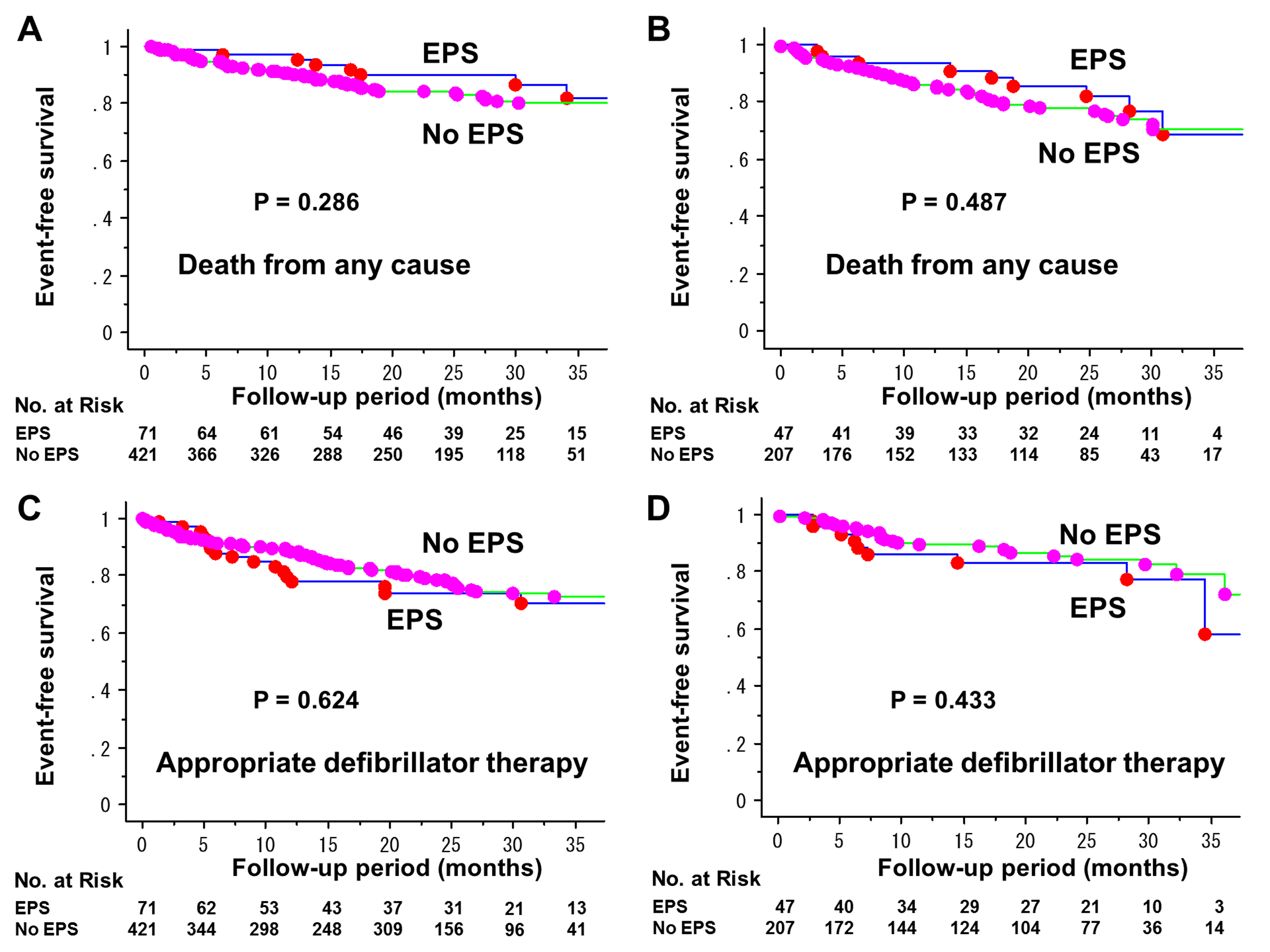

Supplement: Supplementary file 1 — Fig S1 [file JOA3-37-148-s001.tif]

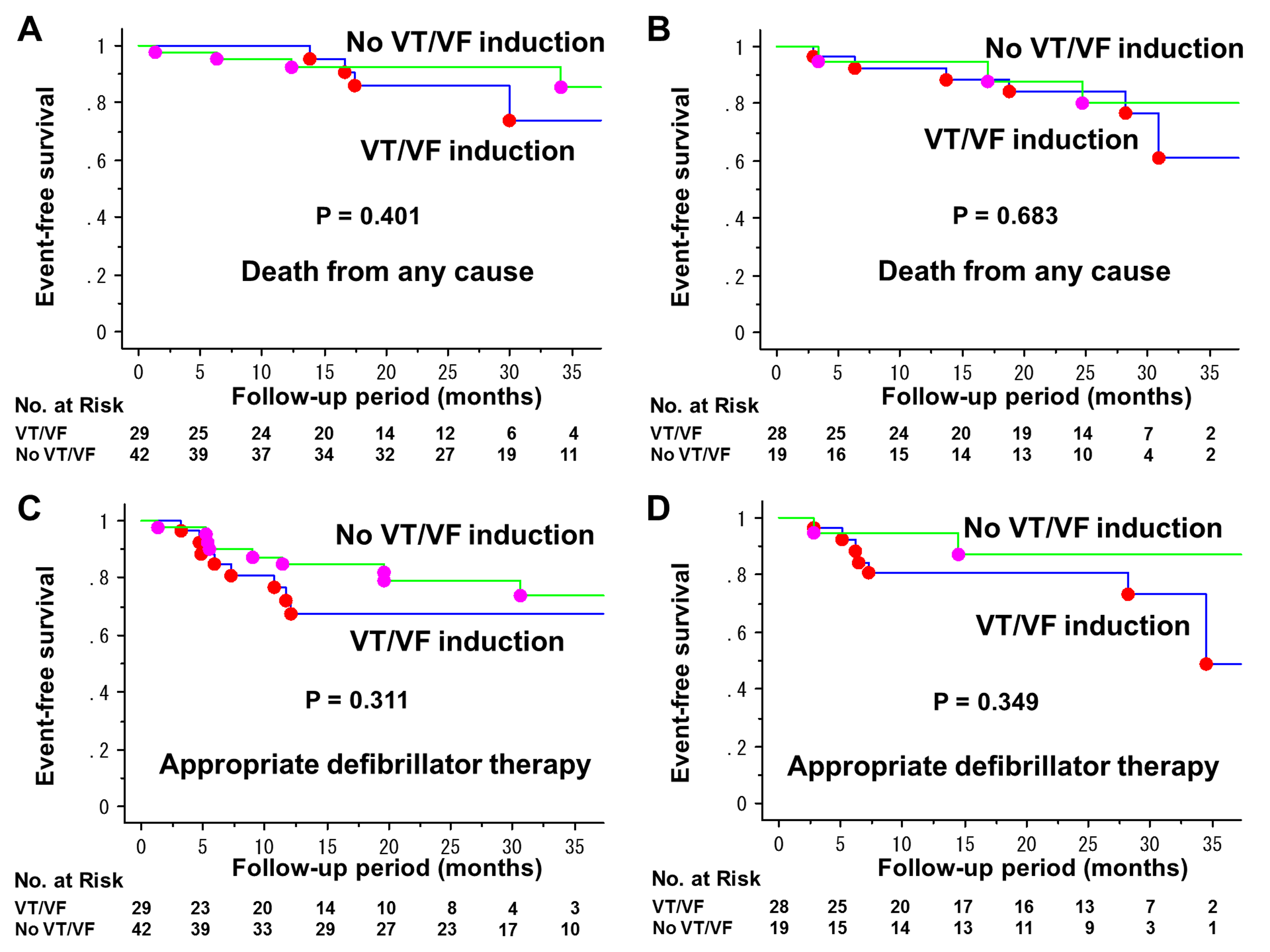

Supplement: Supplementary file 2 — Fig S2 [file JOA3-37-148-s002.tif]
